# C—H⋯π packing inter­actions in 2-[5,5-bis­(4-benzyl­oxyphen­yl)-3-cyano-4-methyl-2,5-dihydro­furan-2-yl­idene]malononitrile

**DOI:** 10.1107/S1600536811043480

**Published:** 2011-10-29

**Authors:** Graeme J. Gainsford, Jack Anderson, M. Delower H. Bhuiyan, Andrew J. Kay

**Affiliations:** a Industrial Research Limited, PO Box 31-310, Lower Hutt, New Zealand

## Abstract

The title mol­ecule, C_35_H_25_N_3_O_3_, packs utilizing C—H⋯π attractive inter­actions causing the identical 4-benzyl­oxyphenyl groups to pack with different conformational angles. This difference is consistent with the variable inter­planar dihedral angles found in closely related structures.

## Related literature

For general background, see: Smith *et al.* (2006 ▶, 2010 ▶); Teshome *et al.* (2009 ▶); Datta & Pati (2003 ▶). For related structures, see: Li *et al.* (2005 ▶); Nikitin *et al.* (2010 ▶); Roesky *et al.* (1997 ▶); Wang *et al.* (2007 ▶); Gainsford *et al.* (2008 ▶). For synthesis details, see: Anderson (2009 ▶). For C—H⋯π bonding, see: Desiraju & Steiner (1999 ▶).
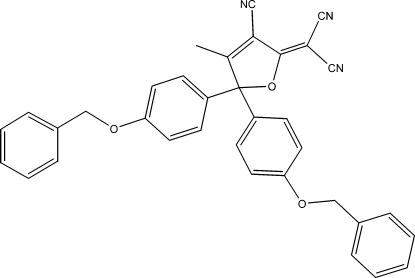


## Experimental

### 

#### Crystal data

C_35_H_25_N_3_O_3_
*M* *_r_* = 535.58
Monoclinic, 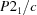

*a* = 18.1696 (8) Å
*b* = 10.0728 (5) Å
*c* = 15.8413 (7) Å
β = 103.779 (3)°
*V* = 2815.8 (2) Å^3^
*Z* = 4
Mo *K*α radiation
μ = 0.08 mm^−1^
*T* = 123 K
0.35 × 0.21 × 0.09 mm

#### Data collection

Bruker–Nonius APEXII CCD area-detector diffractometer
Absorption correction: multi-scan (*SADABS*; Blessing, 1995 ▶; Bruker, 2005 ▶) *T*_min_ = 0.664, *T*_max_ = 0.746
62528 measured reflections
7017 independent reflections
4764 reflections with *I* > 2σ(*I*)
*R*_int_ = 0.073

#### Refinement

*R*[*F* ^2^ > 2σ(*F* ^2^)] = 0.047
*wR*(*F* ^2^) = 0.115
*S* = 1.05
7017 reflections
372 parameters
H-atom parameters constrained
Δρ_max_ = 0.26 e Å^−3^
Δρ_min_ = −0.19 e Å^−3^

### 

Data collection: *APEX2* (Bruker, 2005 ▶); cell refinement: *SAINT* (Bruker, 2005 ▶); data reduction: *SAINT*; program(s) used to solve structure: *SHELXS97* (Sheldrick, 2008 ▶); program(s) used to refine structure: *SHELXL97* (Sheldrick, 2008 ▶); molecular graphics: *ORTEP-3* (Farrugia, 1997 ▶) and *Mercury* (Macrae *et al.*, 2008 ▶); software used to prepare material for publication: *SHELXL97* and *PLATON* (Spek, 2009 ▶).

## Supplementary Material

Crystal structure: contains datablock(s) global, I. DOI: 10.1107/S1600536811043480/bg2427sup1.cif

Structure factors: contains datablock(s) I. DOI: 10.1107/S1600536811043480/bg2427Isup2.hkl

Supplementary material file. DOI: 10.1107/S1600536811043480/bg2427Isup3.cml

Additional supplementary materials:  crystallographic information; 3D view; checkCIF report

## Figures and Tables

**Table 1:** Hydrogen-bond geometry (Å, °) *Cg*1–3 represent the centroids of the phenyl rings C10–C15, C17–C22 and C23–C28, respectively.

*D*—H⋯*A*	*D*—H	H⋯*A*	*D*⋯*A*	*D*—H⋯*A*
C20—H20⋯*Cg*1^i^	0.95	2.85	3.596 (2)	136
C29—-H29*A*⋯*Cg*1^ii^	0.99	2.59	3.5276 (17)	158
C33—-H33⋯*Cg*2^iii^	0.95	2.77	3.697 (3)	167
C16—-H16*B*⋯*Cg*3^iv^	0.99	2.97	3.9262 (18)	162

Symmetry codes: (i) 
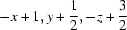
; (ii) 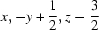; (iii) 
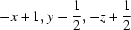
; (iv) 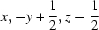.

**Fig. 1.**
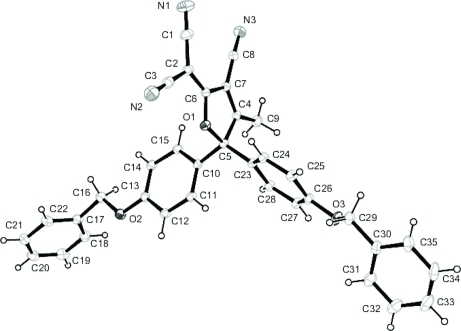
Molecular structure of the asymmetric unit (Farrugia, 1997); displacement ellipsoids are shown at the 30% probability level.

**Fig. 2.**
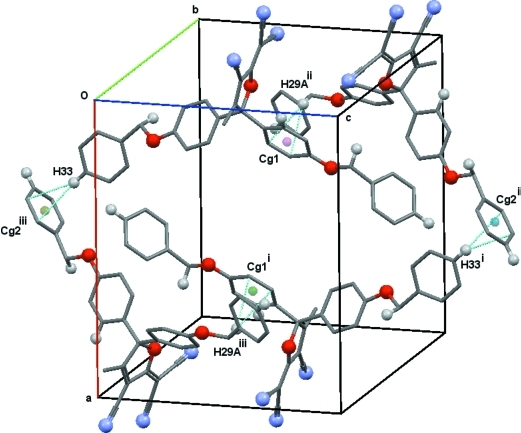
Packing diagram [Mercury, Macrae et al.,(2008)] of the unit cell. Non-hydrogen atoms, ring centroids (Cg) and H atoms involved in C–H···π bonding shown as balls: two close contacts indicated by dotted lines identify the bonds (see text). Symmetry (i) 1 - x, 1 - y, 1 - z (ii) x, 1.5 - y, 1/2 + z (iii) 1 - x, y - 1/2, 1/2 - z.

## supplementary crystallographic information

### Comment

New electro-optic modulators will be key components for high capacity transmissions in the telecommunications industry. Organic nonlinear optical (NLO) chromophores appear to offer a very attractive alternative to currently used inorganic materials (*e.g.* LiNbO3) as they have a much faster response times, are easer to prepare, have low drive voltages and low signal losses. However, issues of aggregation, photochemical & thermal stability are proving significant barriers to the successful uptake of organic NLO materials. A considerable effort has been made over the last two decades to develop organic chromophores with the largest possible NLO response. Due to their dipolar nature, strong electrostatic interactions are possible between individual NLO chromophore molecules which leads to a significant tendency to aggregate (Smith *et al.*, 2006; Datta & Pati, 2003; Teshome *et al.*, 2009). Therefore, much effort has been expended developing methods to minimize aggregation between NLO chromophores(Smith *et al.*, 2010).

One of the most successful strategies to minimize aggregation has been to add bulky pendant groups onto the chromophores. If the pendant groups are aromatic in nature, stacking interactions between the aromatic rings may result, which can overcome the dipole-dipole interactions that cause aggregation (Smith *et al.*, 2010). We have synthesized a new acceptor (the title compound) with bulky groups to reduce aggregation as well as reduce, or even eliminate rotation around the conjugated polyene bridge - acceptor bond.

The asymmetric unit contents of the title compound(I) are shown in Figure 1. The 5-membered ring plane of atoms O1,C4—C7 (hereafter "CDFP", [3-cyano-5,5-dimethyl-2,5-dihydrofuran-2-ylidene]propanedinitrile) can be regarded as planar with maximum out of plane deviation for O1 of 0.023 (1) Å. The dicyano group (N1,C1,C2,C3,N2,C6) is planar but twisted by 5.32 (10) ° with respect to the "CDFP" group; this is similar to the twist in related compound NOJKUT (Gainsford *et al.*, 2008) of 5.69 (17) °. Atom C5 is essentially tetrahedral with the C23–C5–C10 angle widened to 115.83 (11) ° and the internal O1–C5–C4 102.11 (10) °. The phenyl rings are either close to or statistically planar (*e.g.* ring C17–C22, maximum deviation C19, 0.007 (2) Å). The mean planes of the phenyl groups bound directly to the CDFP atom C5 (C10–C15, C23–C28) make angles of 88.70 (8) & 67.60 (8) ° to the CDFP plane and 69.84 (7) ° to each other. This last value is similar to those observed in 1,1,1-tris(4-benzyloxyphenyl)ethane 78.26 (17), 88.89 (17) & 86.27 (18) ° (GERLIY, Roesky *et al.*, 1997), 2,2-bis(4-(benzoyloxy)phenyl)propane 80.3 (1) ° (KIKKAR, Wang *et al.*, 2007) and bis(4-(benzyloxy)phenyl)methane 84.9 (2) & 81.5 (2) ° (SUHNEP, Nikitin *et al.*, 2010). (Compound REFCODES are from the C.S.D., Version 5.32, with August 2011 updates; Allen, 2002).

The main difference observed in the structure is in the relative angular dispositions of the terminal phenyl groups. Here significant differences are seen with the different angles to their attached phenyl rings: 88.79 ° (C17–C22) and 37.81 (8) ° (C30–C35) respectively. It is only after consideration of the molecular packing that this deviation for the pendant identical chemical groups can be rationalized. The crystal packing is dominated by C–H···π bonds (no other significant interactions are observed) with the strongest interaction involving the methylene hydrogen on C29 (H29A) and the phenyl hydrogen H33 (Table 1, Figure 2). The normal expectation for linked biphenyl rings is for their dihedral angles to be ~90 ° to alleviate adjacent ring H···H interactions. Here the restricted twist (\sim 38 °) noted for just one of the ligand arms (involving C23–C28 & C30–C35 rings) ensures optimal C—H···π attractive overlap between glide plane related molecules.

The benzoyloxy-phenyl ring dihedral angles in the comparable structures, whilst variable, are reasonably consistent with the above analysis. For KIKKAR, the angle is 76.54 (9) ° with one C—H···π interaction involving the methylene hydrogen and for SUHNEP, 10.4 (2) & 8.6 (2) ° with six C–H···π interactions utilizing methylene & phenyl hydrogen atoms. Finally for GERLIY the angles are 78.9 (2), 7.7 (2) and 30.6 (2) ° with two C–H···π interactions involving one methylene and one phenyl hydrogen. Apparently, the orientation of the terminal benzoylozy groups with respect to the attached phenyl group is highly dependent on the C–H···π interactions, so no strict orientation rule can be defined.

There are other intermolecular interactions in (I) (Table 1) but the two highlighted (Figure 2 and above) are both closer (C_g_···H < 2.8 Å) and have maximized C–H···C_g_ angles (Desiraju & Steiner, 1999). In Table 1 & Figure 2, labels *Cg*1–3 represent the centroids of the phenyl rings C10–C15, C17–C22 & C23–C28 respectively. In conclusion, we note that C–H···π interactions add to the list of weak but important interactions in crystal formation, so that the preferred molecular alignment of the target molecules is not attained.

### Experimental

Compound(I) was prepared by the condensation of 1,1-bis(4-(benzyloxy)phenyl)-1-hydroxypropan-2-one with 4 equivalents of malononitrile over 10 days as described in Anderson (2009). Crystals were obtained from a 1:1 dichloromethane:acetone mixture.

### Refinement

Five reflections affected by the backstop and 6 others which were clearly outlier data (also at low angle) were omitted from the refinements (using *OMIT*). The methyl and other H atoms were refined with *U*_iso_ 1.5 & 1.2 times respectively that of the *U*_eq_ of their parent atom. All H atoms bound to carbon were constrained to their expected geometries (C—H 0.95, 0.98 & 0.99 Å).

### Crystal data

**Table:**

C_35_H_25_N_3_O_3_	*F*(000) = 1120
*M**_r_* = 535.58	*D*_x_ = 1.263 Mg m^−^^3^
Monoclinic, *P*2_1_/*c*	Mo *K*α radiation, λ = 0.71073 Å
Hall symbol: -P 2ybc	Cell parameters from 7358 reflections
*a* = 18.1696 (8) Å	θ = 2.3–26.7°
*b* = 10.0728 (5) Å	µ = 0.08 mm^−^^1^
*c* = 15.8413 (7) Å	*T* = 123 K
β = 103.779 (3)°	Triangular, pink
*V* = 2815.8 (2) Å^3^	0.35 × 0.21 × 0.09 mm
*Z* = 4	

### Data collection

**Table:**

Bruker–Nonius APEXII CCD area-detector diffractometer	7017 independent reflections
Radiation source: fine-focus sealed tube	4764 reflections with *I* > 2σ(*I*)
graphite	*R*_int_ = 0.073
Detector resolution: 8.333 pixels mm^-1^	θ_max_ = 28.5°, θ_min_ = 2.6°
φ and ω scans	*h* = −24→24
Absorption correction: multi-scan (Software?; Blessing, 1995)	*k* = −13→13
*T*_min_ = 0.664, *T*_max_ = 0.746	*l* = −21→21
62528 measured reflections	

### Refinement

**Table:**

Refinement on *F*^2^	Secondary atom site location: difference Fourier map
Least-squares matrix: full	Hydrogen site location: inferred from neighbouring sites
*R*[*F*^2^ > 2σ(*F*^2^)] = 0.047	H-atom parameters constrained
*wR*(*F*^2^) = 0.115	*w* = 1/[σ^2^(*F*_o_^2^) + (0.0408*P*)^2^ + 0.6136*P*] where *P* = (*F*_o_^2^ + 2*F*_c_^2^)/3
*S* = 1.05	(Δ/σ)_max_ < 0.001
7017 reflections	Δρ_max_ = 0.26 e Å^−^^3^
372 parameters	Δρ_min_ = −0.19 e Å^−^^3^
0 restraints	Extinction correction: *SHELXL97* (Sheldrick, 2008), Fc^*^=kFc[1+0.001xFc^2^λ^3^/sin(2θ)]^-1/4^
Primary atom site location: structure-invariant direct methods	Extinction coefficient: 0.0023 (5)

### Special details

**Table:**

Geometry. All e.s.d.'s (except the e.s.d. in the dihedral angle between two l.s. planes) are estimated using the full covariance matrix. The cell e.s.d.'s are taken into account individually in the estimation of e.s.d.'s in distances, angles and torsion angles; correlations between e.s.d.'s in cell parameters are only used when they are defined by crystal symmetry. An approximate (isotropic) treatment of cell e.s.d.'s is used for estimating e.s.d.'s involving l.s. planes.
Refinement. Refinement of *F*^2^ against ALL reflections. The weighted *R*-factor *wR* and goodness of fit *S* are based on *F*^2^, conventional *R*-factors *R* are based on *F*, with *F* set to zero for negative *F*^2^. The threshold expression of *F*^2^ > σ(*F*^2^) is used only for calculating *R*-factors(gt) *etc*. and is not relevant to the choice of reflections for refinement. *R*-factors based on *F*^2^ are statistically about twice as large as those based on *F*, and *R*- factors based on ALL data will be even larger.

### Fractional atomic coordinates and isotropic or equivalent isotropic displacement parameters (Å^2^)

**Table:**

	*x*	*y*	*z*	*U*_iso_*/*U*_eq_	
O1	0.12002 (5)	0.68767 (10)	0.34643 (6)	0.0281 (2)	
O2	0.39969 (6)	0.75816 (11)	0.64285 (6)	0.0345 (3)	
O3	0.25635 (6)	0.44822 (11)	0.04549 (6)	0.0378 (3)	
N1	−0.12627 (9)	0.69465 (18)	0.39894 (12)	0.0612 (5)	
N2	0.02878 (9)	0.99105 (17)	0.32599 (10)	0.0516 (4)	
N3	−0.05524 (8)	0.37366 (16)	0.41384 (10)	0.0507 (4)	
C1	−0.07014 (10)	0.71929 (18)	0.38148 (11)	0.0423 (4)	
C2	0.00032 (8)	0.74932 (16)	0.36127 (9)	0.0325 (3)	
C3	0.01570 (9)	0.88387 (18)	0.34204 (10)	0.0374 (4)	
C4	0.12121 (8)	0.46134 (15)	0.37889 (9)	0.0279 (3)	
C5	0.17157 (8)	0.57042 (14)	0.35864 (9)	0.0259 (3)	
C6	0.05397 (8)	0.65399 (15)	0.36280 (9)	0.0277 (3)	
C7	0.05349 (8)	0.51294 (15)	0.38106 (9)	0.0285 (3)	
C8	−0.00822 (9)	0.43825 (16)	0.39840 (10)	0.0350 (4)	
C9	0.14590 (10)	0.32107 (15)	0.39324 (11)	0.0385 (4)	
H9A	0.1042	0.2675	0.4045	0.058*	
H9B	0.1895	0.3157	0.4433	0.058*	
H9C	0.1603	0.2873	0.3414	0.058*	
C10	0.23631 (8)	0.60639 (14)	0.43524 (9)	0.0256 (3)	
C11	0.30259 (8)	0.66571 (15)	0.42418 (9)	0.0290 (3)	
H11	0.3107	0.6735	0.3673	0.035*	
C12	0.35666 (8)	0.71334 (15)	0.49400 (9)	0.0305 (3)	
H12	0.4020	0.7517	0.4852	0.037*	
C13	0.34456 (8)	0.70514 (15)	0.57738 (9)	0.0290 (3)	
C14	0.27911 (8)	0.64498 (15)	0.58974 (9)	0.0306 (3)	
H14	0.2708	0.6379	0.6465	0.037*	
C15	0.22617 (8)	0.59547 (15)	0.51933 (9)	0.0293 (3)	
H15	0.1820	0.5531	0.5285	0.035*	
C16	0.38713 (9)	0.74530 (18)	0.72893 (9)	0.0399 (4)	
H16A	0.3792	0.6507	0.7413	0.048*	
H16B	0.3410	0.7951	0.7326	0.048*	
C17	0.45409 (9)	0.79826 (16)	0.79476 (9)	0.0332 (4)	
C18	0.51398 (10)	0.71623 (18)	0.83300 (10)	0.0405 (4)	
H18	0.5142	0.6262	0.8152	0.049*	
C19	0.57374 (10)	0.76491 (19)	0.89730 (11)	0.0444 (4)	
H19	0.6151	0.7087	0.9226	0.053*	
C20	0.57305 (10)	0.89412 (19)	0.92428 (11)	0.0432 (4)	
H20	0.6135	0.9268	0.9690	0.052*	
C21	0.51405 (9)	0.97639 (19)	0.88694 (11)	0.0441 (4)	
H21	0.5137	1.0660	0.9055	0.053*	
C22	0.45512 (9)	0.92836 (17)	0.82220 (11)	0.0386 (4)	
H22	0.4146	0.9859	0.7962	0.046*	
C23	0.19339 (8)	0.54421 (14)	0.27323 (9)	0.0258 (3)	
C24	0.15600 (8)	0.60416 (15)	0.19629 (9)	0.0285 (3)	
H24	0.1167	0.6662	0.1968	0.034*	
C25	0.17488 (8)	0.57509 (15)	0.11830 (9)	0.0305 (3)	
H25	0.1488	0.6172	0.0661	0.037*	
C26	0.23175 (8)	0.48458 (15)	0.11714 (9)	0.0288 (3)	
C27	0.26916 (8)	0.42278 (15)	0.19397 (9)	0.0306 (3)	
H27	0.3078	0.3596	0.1932	0.037*	
C28	0.25050 (8)	0.45259 (15)	0.27101 (9)	0.0292 (3)	
H28	0.2767	0.4104	0.3232	0.035*	
C29	0.22598 (9)	0.51569 (16)	−0.03469 (9)	0.0341 (4)	
H29A	0.2287	0.6130	−0.0258	0.041*	
H29B	0.1723	0.4905	−0.0581	0.041*	
C30	0.27319 (10)	0.47451 (15)	−0.09672 (10)	0.0356 (4)	
C31	0.35127 (10)	0.46188 (17)	−0.06759 (11)	0.0431 (4)	
H31	0.3750	0.4812	−0.0087	0.052*	
C32	0.39502 (12)	0.42104 (19)	−0.12404 (13)	0.0551 (5)	
H32	0.4483	0.4109	−0.1035	0.066*	
C33	0.36105 (14)	0.39554 (19)	−0.20918 (14)	0.0607 (6)	
H33	0.3909	0.3671	−0.2476	0.073*	
C34	0.28422 (15)	0.41071 (18)	−0.23958 (12)	0.0569 (6)	
H34	0.2612	0.3942	−0.2991	0.068*	
C35	0.23988 (12)	0.45034 (16)	−0.18336 (10)	0.0447 (4)	
H35	0.1867	0.4608	−0.2046	0.054*	

### Atomic displacement parameters (Å^2^)

**Table:**

	*U*^11^	*U*^22^	*U*^33^	*U*^12^	*U*^13^	*U*^23^
O1	0.0257 (5)	0.0299 (6)	0.0299 (5)	0.0039 (4)	0.0088 (4)	0.0006 (4)
O2	0.0341 (6)	0.0465 (7)	0.0231 (5)	−0.0072 (5)	0.0073 (4)	−0.0020 (5)
O3	0.0513 (7)	0.0409 (6)	0.0252 (5)	0.0108 (5)	0.0173 (5)	0.0047 (5)
N1	0.0379 (9)	0.0759 (12)	0.0742 (12)	0.0119 (8)	0.0223 (8)	0.0099 (10)
N2	0.0505 (10)	0.0466 (10)	0.0561 (10)	0.0130 (8)	0.0097 (8)	0.0069 (8)
N3	0.0436 (9)	0.0545 (10)	0.0581 (10)	−0.0150 (7)	0.0205 (8)	−0.0137 (8)
C1	0.0325 (9)	0.0510 (11)	0.0433 (10)	0.0102 (8)	0.0092 (8)	0.0017 (8)
C2	0.0268 (8)	0.0419 (9)	0.0285 (8)	0.0048 (7)	0.0058 (6)	−0.0011 (7)
C3	0.0327 (9)	0.0458 (11)	0.0328 (8)	0.0135 (8)	0.0061 (7)	0.0010 (8)
C4	0.0312 (8)	0.0313 (8)	0.0221 (7)	−0.0023 (6)	0.0080 (6)	−0.0032 (6)
C5	0.0253 (7)	0.0266 (7)	0.0262 (7)	0.0045 (6)	0.0072 (6)	0.0011 (6)
C6	0.0236 (7)	0.0390 (9)	0.0199 (7)	0.0000 (6)	0.0039 (6)	−0.0036 (6)
C7	0.0277 (8)	0.0344 (8)	0.0239 (7)	−0.0037 (6)	0.0075 (6)	−0.0051 (6)
C8	0.0317 (8)	0.0403 (9)	0.0340 (8)	−0.0057 (7)	0.0098 (7)	−0.0103 (7)
C9	0.0450 (10)	0.0301 (9)	0.0446 (9)	−0.0006 (7)	0.0187 (8)	−0.0012 (7)
C10	0.0270 (7)	0.0257 (7)	0.0247 (7)	0.0038 (6)	0.0072 (6)	0.0010 (6)
C11	0.0308 (8)	0.0346 (8)	0.0236 (7)	0.0013 (6)	0.0102 (6)	0.0019 (6)
C12	0.0285 (8)	0.0364 (9)	0.0281 (8)	−0.0019 (6)	0.0095 (6)	0.0020 (6)
C13	0.0297 (8)	0.0311 (8)	0.0254 (7)	0.0010 (6)	0.0049 (6)	−0.0010 (6)
C14	0.0338 (8)	0.0364 (8)	0.0236 (7)	−0.0007 (7)	0.0110 (6)	0.0011 (6)
C15	0.0298 (8)	0.0334 (8)	0.0266 (7)	−0.0014 (6)	0.0104 (6)	0.0013 (6)
C16	0.0417 (9)	0.0539 (11)	0.0251 (8)	−0.0118 (8)	0.0099 (7)	−0.0017 (7)
C17	0.0340 (8)	0.0436 (9)	0.0236 (7)	−0.0051 (7)	0.0096 (6)	0.0020 (7)
C18	0.0475 (10)	0.0412 (10)	0.0345 (9)	−0.0012 (8)	0.0129 (8)	−0.0008 (7)
C19	0.0405 (10)	0.0561 (12)	0.0355 (9)	0.0050 (8)	0.0069 (8)	0.0136 (8)
C20	0.0382 (9)	0.0602 (12)	0.0299 (8)	−0.0104 (8)	0.0053 (7)	−0.0005 (8)
C21	0.0400 (10)	0.0454 (10)	0.0468 (10)	−0.0088 (8)	0.0105 (8)	−0.0091 (8)
C22	0.0315 (8)	0.0429 (10)	0.0407 (9)	−0.0007 (7)	0.0074 (7)	0.0038 (7)
C23	0.0243 (7)	0.0286 (8)	0.0252 (7)	−0.0011 (6)	0.0072 (6)	−0.0012 (6)
C24	0.0276 (7)	0.0307 (8)	0.0277 (7)	0.0032 (6)	0.0077 (6)	0.0014 (6)
C25	0.0331 (8)	0.0342 (8)	0.0237 (7)	0.0024 (7)	0.0058 (6)	0.0047 (6)
C26	0.0342 (8)	0.0292 (8)	0.0252 (7)	−0.0016 (6)	0.0115 (6)	−0.0008 (6)
C27	0.0319 (8)	0.0306 (8)	0.0308 (8)	0.0046 (6)	0.0104 (6)	0.0003 (6)
C28	0.0298 (8)	0.0325 (8)	0.0254 (7)	0.0035 (6)	0.0069 (6)	0.0025 (6)
C29	0.0451 (9)	0.0335 (9)	0.0242 (7)	−0.0003 (7)	0.0090 (7)	0.0017 (6)
C30	0.0560 (11)	0.0253 (8)	0.0284 (8)	−0.0037 (7)	0.0157 (7)	0.0010 (6)
C31	0.0571 (11)	0.0409 (10)	0.0354 (9)	0.0058 (8)	0.0189 (8)	0.0040 (7)
C32	0.0688 (13)	0.0478 (11)	0.0602 (13)	0.0076 (10)	0.0379 (11)	0.0078 (9)
C33	0.0989 (18)	0.0425 (11)	0.0569 (13)	−0.0029 (11)	0.0507 (13)	−0.0045 (9)
C34	0.1087 (18)	0.0387 (10)	0.0301 (9)	−0.0159 (11)	0.0301 (11)	−0.0068 (8)
C35	0.0714 (13)	0.0338 (9)	0.0304 (8)	−0.0126 (8)	0.0150 (8)	−0.0014 (7)

### Geometric parameters (Å, °)

**Table:**

O1—C6	1.3307 (16)	C17—C22	1.379 (2)
O1—C5	1.4911 (16)	C17—C18	1.386 (2)
O2—C13	1.3673 (17)	C18—C19	1.389 (2)
O2—C16	1.4410 (17)	C18—H18	0.9500
O3—C26	1.3654 (16)	C19—C20	1.371 (3)
O3—C29	1.4303 (17)	C19—H19	0.9500
N1—C1	1.146 (2)	C20—C21	1.372 (2)
N2—C3	1.147 (2)	C20—H20	0.9500
N3—C8	1.146 (2)	C21—C22	1.382 (2)
C1—C2	1.424 (2)	C21—H21	0.9500
C2—C6	1.364 (2)	C22—H22	0.9500
C2—C3	1.431 (2)	C23—C24	1.385 (2)
C4—C7	1.344 (2)	C23—C28	1.395 (2)
C4—C9	1.484 (2)	C24—C25	1.3899 (19)
C4—C5	1.512 (2)	C24—H24	0.9500
C5—C10	1.5190 (19)	C25—C26	1.381 (2)
C5—C23	1.5208 (19)	C25—H25	0.9500
C6—C7	1.450 (2)	C26—C27	1.392 (2)
C7—C8	1.430 (2)	C27—C28	1.3755 (19)
C9—H9A	0.9800	C27—H27	0.9500
C9—H9B	0.9800	C28—H28	0.9500
C9—H9C	0.9800	C29—C30	1.508 (2)
C10—C15	1.3919 (19)	C29—H29A	0.9900
C10—C11	1.392 (2)	C29—H29B	0.9900
C11—C12	1.379 (2)	C30—C35	1.383 (2)
C11—H11	0.9500	C30—C31	1.389 (2)
C12—C13	1.3921 (19)	C31—C32	1.393 (2)
C12—H12	0.9500	C31—H31	0.9500
C13—C14	1.390 (2)	C32—C33	1.367 (3)
C14—C15	1.381 (2)	C32—H32	0.9500
C14—H14	0.9500	C33—C34	1.372 (3)
C15—H15	0.9500	C33—H33	0.9500
C16—C17	1.499 (2)	C34—C35	1.394 (3)
C16—H16A	0.9900	C34—H34	0.9500
C16—H16B	0.9900	C35—H35	0.9500
			
C6—O1—C5	109.92 (11)	C17—C18—C19	120.35 (17)
C13—O2—C16	115.48 (11)	C17—C18—H18	119.8
C26—O3—C29	118.50 (12)	C19—C18—H18	119.8
N1—C1—C2	179.0 (2)	C20—C19—C18	120.00 (16)
C6—C2—C1	121.55 (15)	C20—C19—H19	120.0
C6—C2—C3	119.70 (14)	C18—C19—H19	120.0
C1—C2—C3	118.72 (14)	C19—C20—C21	120.21 (16)
N2—C3—C2	179.00 (18)	C19—C20—H20	119.9
C7—C4—C9	127.58 (14)	C21—C20—H20	119.9
C7—C4—C5	109.18 (13)	C20—C21—C22	119.74 (17)
C9—C4—C5	123.24 (13)	C20—C21—H21	120.1
O1—C5—C4	102.11 (10)	C22—C21—H21	120.1
O1—C5—C10	104.93 (11)	C17—C22—C21	121.10 (16)
C4—C5—C10	113.35 (11)	C17—C22—H22	119.5
O1—C5—C23	108.11 (11)	C21—C22—H22	119.5
C4—C5—C23	111.21 (11)	C24—C23—C28	118.56 (13)
C10—C5—C23	115.83 (11)	C24—C23—C5	122.02 (12)
O1—C6—C2	119.39 (14)	C28—C23—C5	119.36 (12)
O1—C6—C7	109.70 (12)	C23—C24—C25	121.15 (13)
C2—C6—C7	130.90 (14)	C23—C24—H24	119.4
C4—C7—C8	124.47 (14)	C25—C24—H24	119.4
C4—C7—C6	108.95 (13)	C26—C25—C24	119.65 (13)
C8—C7—C6	126.57 (13)	C26—C25—H25	120.2
N3—C8—C7	176.72 (19)	C24—C25—H25	120.2
C4—C9—H9A	109.5	O3—C26—C25	125.61 (13)
C4—C9—H9B	109.5	O3—C26—C27	114.74 (13)
H9A—C9—H9B	109.5	C25—C26—C27	119.65 (13)
C4—C9—H9C	109.5	C28—C27—C26	120.39 (14)
H9A—C9—H9C	109.5	C28—C27—H27	119.8
H9B—C9—H9C	109.5	C26—C27—H27	119.8
C15—C10—C11	118.03 (13)	C27—C28—C23	120.59 (13)
C15—C10—C5	119.46 (12)	C27—C28—H28	119.7
C11—C10—C5	122.02 (12)	C23—C28—H28	119.7
C12—C11—C10	121.38 (13)	O3—C29—C30	106.78 (13)
C12—C11—H11	119.3	O3—C29—H29A	110.4
C10—C11—H11	119.3	C30—C29—H29A	110.4
C11—C12—C13	119.81 (13)	O3—C29—H29B	110.4
C11—C12—H12	120.1	C30—C29—H29B	110.4
C13—C12—H12	120.1	H29A—C29—H29B	108.6
O2—C13—C14	124.09 (13)	C35—C30—C31	118.96 (16)
O2—C13—C12	116.35 (13)	C35—C30—C29	120.83 (16)
C14—C13—C12	119.56 (13)	C31—C30—C29	120.20 (14)
C15—C14—C13	119.91 (13)	C30—C31—C32	120.51 (17)
C15—C14—H14	120.0	C30—C31—H31	119.7
C13—C14—H14	120.0	C32—C31—H31	119.7
C14—C15—C10	121.27 (13)	C33—C32—C31	119.8 (2)
C14—C15—H15	119.4	C33—C32—H32	120.1
C10—C15—H15	119.4	C31—C32—H32	120.1
O2—C16—C17	109.94 (12)	C32—C33—C34	120.51 (18)
O2—C16—H16A	109.7	C32—C33—H33	119.7
C17—C16—H16A	109.7	C34—C33—H33	119.7
O2—C16—H16B	109.7	C33—C34—C35	120.14 (18)
C17—C16—H16B	109.7	C33—C34—H34	119.9
H16A—C16—H16B	108.2	C35—C34—H34	119.9
C22—C17—C18	118.58 (15)	C30—C35—C34	120.09 (19)
C22—C17—C16	120.34 (15)	C30—C35—H35	120.0
C18—C17—C16	120.97 (16)	C34—C35—H35	120.0
			
C6—O1—C5—C4	3.74 (14)	C13—O2—C16—C17	175.86 (13)
C6—O1—C5—C10	−114.73 (12)	O2—C16—C17—C22	93.34 (17)
C6—O1—C5—C23	121.10 (12)	O2—C16—C17—C18	−90.50 (18)
C7—C4—C5—O1	−2.30 (14)	C22—C17—C18—C19	−0.3 (2)
C9—C4—C5—O1	177.55 (13)	C16—C17—C18—C19	−176.51 (14)
C7—C4—C5—C10	110.01 (13)	C17—C18—C19—C20	1.1 (2)
C9—C4—C5—C10	−70.14 (17)	C18—C19—C20—C21	−1.1 (2)
C7—C4—C5—C23	−117.41 (13)	C19—C20—C21—C22	0.2 (3)
C9—C4—C5—C23	62.44 (18)	C18—C17—C22—C21	−0.6 (2)
C5—O1—C6—C2	175.49 (12)	C16—C17—C22—C21	175.68 (15)
C5—O1—C6—C7	−3.83 (15)	C20—C21—C22—C17	0.6 (2)
C1—C2—C6—O1	−177.65 (13)	O1—C5—C23—C24	−12.82 (18)
C3—C2—C6—O1	0.1 (2)	C4—C5—C23—C24	98.50 (16)
C1—C2—C6—C7	1.5 (3)	C10—C5—C23—C24	−130.18 (14)
C3—C2—C6—C7	179.23 (14)	O1—C5—C23—C28	170.13 (12)
C9—C4—C7—C8	0.1 (2)	C4—C5—C23—C28	−78.55 (16)
C5—C4—C7—C8	179.99 (13)	C10—C5—C23—C28	52.77 (18)
C9—C4—C7—C6	−179.62 (14)	C28—C23—C24—C25	−0.5 (2)
C5—C4—C7—C6	0.22 (16)	C5—C23—C24—C25	−177.53 (13)
O1—C6—C7—C4	2.29 (16)	C23—C24—C25—C26	0.2 (2)
C2—C6—C7—C4	−176.92 (15)	C29—O3—C26—C25	5.4 (2)
O1—C6—C7—C8	−177.47 (13)	C29—O3—C26—C27	−174.32 (13)
C2—C6—C7—C8	3.3 (3)	C24—C25—C26—O3	−179.27 (14)
O1—C5—C10—C15	78.88 (15)	C24—C25—C26—C27	0.4 (2)
C4—C5—C10—C15	−31.71 (18)	O3—C26—C27—C28	178.91 (13)
C23—C5—C10—C15	−162.01 (13)	C25—C26—C27—C28	−0.8 (2)
O1—C5—C10—C11	−92.98 (15)	C26—C27—C28—C23	0.6 (2)
C4—C5—C10—C11	156.44 (13)	C24—C23—C28—C27	0.1 (2)
C23—C5—C10—C11	26.14 (19)	C5—C23—C28—C27	177.22 (13)
C15—C10—C11—C12	−0.4 (2)	C26—O3—C29—C30	169.49 (12)
C5—C10—C11—C12	171.53 (13)	O3—C29—C30—C35	140.52 (14)
C10—C11—C12—C13	−1.5 (2)	O3—C29—C30—C31	−40.28 (19)
C16—O2—C13—C14	1.5 (2)	C35—C30—C31—C32	−2.2 (2)
C16—O2—C13—C12	−178.30 (14)	C29—C30—C31—C32	178.57 (15)
C11—C12—C13—O2	−178.06 (13)	C30—C31—C32—C33	1.2 (3)
C11—C12—C13—C14	2.1 (2)	C31—C32—C33—C34	0.4 (3)
O2—C13—C14—C15	179.31 (14)	C32—C33—C34—C35	−1.0 (3)
C12—C13—C14—C15	−0.9 (2)	C31—C30—C35—C34	1.6 (2)
C13—C14—C15—C10	−1.1 (2)	C29—C30—C35—C34	−179.18 (15)
C11—C10—C15—C14	1.7 (2)	C33—C34—C35—C30	0.0 (3)
C5—C10—C15—C14	−170.48 (13)		

### Hydrogen-bond geometry (Å, °)

**Table:**

Cg1–3 represent the centroids of the phenyl rings C10–C15, C17–C22 and C23–C28, respectively.

**Table:**

*D*—H···*A*	*D*—H	H···*A*	*D*···*A*	*D*—H···*A*
C20—H20···Cg1^i^	0.95	2.85	3.596 (2)	136
C29—-H29A···Cg1^ii^	0.99	2.59	3.5276 (17)	158
C33—-H33···Cg2^iii^	0.95	2.77	3.697 (3)	167
C16—-H16B···Cg3^iv^	0.99	2.97	3.9262 (18)	162

Symmetry codes: (i) −*x*+1, *y*+1/2, −*z*+3/2; (ii) *x*, −*y*+1/2, *z*−3/2; (iii) −*x*+1, *y*−1/2, −*z*+1/2; (iv) *x*, −*y*+1/2, *z*−1/2.
